# Management of Small Nonfunctioning Pancreatic Neuroendocrine Neoplasms: Current Opinion and Controversies

**DOI:** 10.3390/jcm12010251

**Published:** 2022-12-29

**Authors:** Woo Hyun Paik, Kyong Joo Lee

**Affiliations:** 1 Department of Internal Medicine, Seoul National University College of Medicine, Seoul 03080, Republic of Korea; 2 Department of Internal Medicine, Hallym University Dongtan Sacred Heart Hospital, Hallym University College of Medicine, Hwaseong 18450, Republic of Korea

**Keywords:** pancreatic neuroendocrine neoplasm, small, asymptomatic, nonfunctional

## Abstract

The incidence of small and asymptomatic pancreatic neuroendocrine neoplasms (PNENs) has increased due to the widespread use of high-resolution diagnostic imaging in screening programs. Most PNENs are slow-growing indolent neoplasms. However, a local invasion or metastasis can sometimes occur with PNENs, leading to a poor prognosis. The management of small, nonfunctioning PNENs remains under debate. The National Comprehensive Cancer Network guidelines recommend observation in selected cases of small PNENs less than 2 cm. Pancreatic surgery remains a high-risk operation with a 28–30% morbidity and 1% mortality. Therefore, the decision on how to manage small PNENs is challenging. This review focuses on the management of small nonfunctioning PNENs. We also highlight the malignant potential of small PNENs according to tumor size, tumor grade, and tumor biomarker. Endoscopic-ultrasound-guided biopsy is recommended to evaluate the potential risk of malignancy. Furthermore, we discuss the current guidelines and future directions for the management of small PNENs.

## 1. Introduction

Pancreatic neuroendocrine neoplasms (PNENs) are relatively rare, accounting for 1–2% of pancreatic malignancies [1]. Recently, improved diagnostic technology increased the incidence of diagnosed PNENs [2]. PNENs can be functional and nonfunctional (NF), as symptoms can be expressed depending on hormone secretion. PNENs can secrete hormones such as insulin, gastrin, glucagon, and vasoactive essential peptides, and can cause multiple endocrine and carcinoid syndromes [3]. Compared with functional tumors, NF PNENs account for 75% to 90% of the total [4].

Although 100 years have passed since Oberndorfer first named a carcinoid tumor in 1907, it has been difficult unifying the naming and classification of pathology and clinically heterogeneous diseases [5]. In 2010, the WHO adopted the classification proposed by the European Neuroendocrine Tumor Society (ENETS) to unify the names as neuroendocrine tumors, and classified them as grade one (G1), grade two (G2), and grade three (G3) using the mitotic count and Ki-67 index [6,7,8]. Generally, G1 and G2 refer to neuroendocrine tumors (NETs), and G3 refers to neuroendocrine carcinoma (NEC). However, recent results have identified two different tumors in G3. Therefore, it was reclassified as NET/NEC, with well and poorly differentiated cell differentiations in the WHO classification in 2017 (Table 1) [9]. The WHO 2022 classification was recently introduced for neuroendocrine neoplasms, and the classification remained the same [10].

The standard treatment for PNENs with these various characteristics is still radical resection, but in the case of small tumors less than 2 cm in size, surgery can be withheld, and the patient is followed-up with. However, there is still not enough evidence to determine the treatment policy for these small tumors. This paper describes the controversial management guidelines for small NF PNENs in clinical practice.

## 2. Malignant Potential of Small PNENs

### 2.1. Tumor Size

Tumor size is an important prognostic factor in predicting the malignant risk of PNENs, and the ENETS consensus guideline reports that only 6% of PNENs below 2 cm are found accidentally [11,12]. In a recent multicenter study in Korea, 118 histologically confirmed cases of small PNENs of 2 cm or less showed malignant potential, such as metastasis or postoperative recurrence, during the follow-up of 45.3 months. The reported incidence of 7% was similar to the ENETS guidelines [13].

Some argue that risk stratification should be further subdivided according to tumor size. In a previous study, in the case of PNENs of less than 1 cm, there were no reported deaths, lymph node metastasis, significant tumor progression, metastasis, or recurrence during the observation period. In these cases, a follow-up observation could be considered a priority instead of surgery [13,14]. Jung et al. showed that 145 small NF PNENs were associated with NET G2 or G3 if the tumor size was 1.5 cm or more if the patient was 55 years old or older, and if the tumor was significantly enlarged (20% or more or 5 mm or more) [15]. It was suggested that if the tumor size was less than 1.5 cm, only a short follow-up was possible in asymptomatic NF PNENs. Kishi et al. reported that in 91 cases involving small PNENs, the tumor was unlikely to worsen if the size was below 1 cm. Still, the risk of metastasis or recurrence increased when the tumor size was over 1.5 cm [16]. In a group involving Chinese patients with NETs, surgery was recommended for patients with small PNENs greater than 1 cm [17]. Therefore, a follow-up observation could be considered first for patients with PNENs less than 1 cm. However, a further evaluation of the malignant potential is required for patients with tumors larger than 1.5 cm.

#### Risk of Lymph Node Metastasis

Several studies have reported on the correlation between tumor size and lymph node metastasis [11,15,18]. Perinel et al. found that for tumors up to 5 cm in size, the likelihood of lymph node metastasis increased by 1.73 for every 1 cm increase in size [19]. A tumor at a size of more than 2 cm was also noted as an additional independent risk factor for lymph node metastasis and disease-free survival [19]. In two large cohort studies of patients with NF PNENs that were less than 2 cm in size, the rate of lymph node metastasis ranged from 27.3% to 29.0%, while the risk of distant metastases ranged from 9.1 to 10.0% [20,21]. According to a multicenter retrospective investigation from Europe, 10% of resected NF PNETs under 2 cm was found to have metastatic lymph nodes [22].

### 2.2. Tumor Grade

In addition to size, a significant factor in predicting the prognosis of patients with PNENs was the WHO grade, with it being more likely that recurrence or metastasis would occur in the future with a higher grade [13,23,24]. In a recent multicenter study in Korea, the proportion of G2 NETs among small PNENs of less than 2 cm was 16.6%. The WHO G2 was the only factor that predicted malignant potential in a multivariate analysis that considered tumor size, imaging characteristics, size change, and the WHO grade (hazard ratio 13.97, 95% confidence interval (CI) 2.60–75.03) [13]. G3 NET/NEC accounts for 1–2% of all PNENs, and the current guidelines recommend radical surgery, including lymphadenectomy. The recommended treatment for G3 NEC is equivalent to that of adenocarcinoma [25]. The WHO grade can be checked in advance with small PNENs using endoscopic-ultrasound (EUS)-guided fine-needle aspiration or biopsy. The ki-67 index showed a high concordance rate in this study by comparing tissues obtained through the EUS-guided fine-needle aspiration test and surgery [26,27]. However, it should be kept in mind that there may be errors with the WHO rating due to tumor heterogeneity, because fine-needle aspiration or biopsy have been used to acquire and analyze very few tumors [27,28]. According to a recent study, the Ki-67 index of cell pathology results and histopathology do not match well. Therefore, it is recommended to use histopathology when evaluating the WHO grade with a fine-needle aspiration test [29].

In addition, the risk of malignancy may increase if the contrast enhancement is not good or is accompanied by a dilatation of the main pancreatic duct in imaging findings [30,31,32,33], especially in the case of small pancreatic tumors with poor contrast enhancement, other malignant or borderline tumors, such as pancreatic adenocarcinoma, solid pseudopapillary tumors, and lymphoma, should be distinguished from PNENs. EUS-guided biopsies should be performed more often because only one-third of cases currently use EUS-guided biopsy when diagnosing PNENs in clinical practice [29,34].

### 2.3. Tumor Biomarker

Chromogranin A (CgA), elevated in 70% of all cases of PNENs, is recognized as a diagnostic marker for all neuroendocrine tumors [35]. CgA has a high sensitivity and good specificity [36,37]. Circulating CgA is correlated with tumor progression, metastasis, and response to treatment in PNENs [38,39]. In a study by Raoof et al., 445 patients with pancreatic neuroendocrine tumors measuring less than 2 cm with no distant metastases were identified and classified as CgA high (>420 ng/mL) or CgA low (≤420 ng/mL) [40]. There were 93 (21%) CgA high patients and 352 (79%) CgA low patients. After adjusting for tumor size, grade, clinical nodal status, and academic status of the facility, CgA levels (CgA high versus CgA low) independently predicted overall survival (hazard ratio: 7.90, 95% CI: 2.34–26.69, *p* = 0.001). Patients in the CgA high subgroup benefited from surgical resection the most (log-rank *p* < 0.001). The tumor aggressiveness of small NF PNENs could be predicted using CgA levels. Therefore, surgical resection should be considered if CgA concentrations were more than 420 ng/mL [40].

Molecular imaging with positron emission tomography/computed tomography (PET/CT) has become a helpful modality in the management of PNENs [41]. Gallium^68^ somatostatin analogues (SSA)-PET and F-18 fluorodeoxyglucose (FDG) PET scans are available in clinical practice. The most used imaging modality in PNENs is the gallium^68^-SSA PET/CT, which is responsible not only for staging and diagnosis, but also for defining the biology of the tumor and its SSA responsiveness [42]. In contrast, the F-18 FDG PET/CT, which is typically useful for predicting the aggressiveness and progression rate of G2–G3 tumors, is not routinely used in all PNENs [43]. The optimum recommended therapeutic approach can be using a combination of gallium^68^-SSA PET/CT and F-18 FDG PET/CT [44].

## 3. Management of Small NF PNENs

### 3.1. Close Follow-Up

In 2012, ENETS presented a “watch-and-wait” procedure for patients with small PNENs of less than 2 cm, which was controversial [12]. The ENETS consensus guidelines recommended a follow-up for patients with small PNENs every three months rather than surgery, and every six months for three years after, considering that only 6% of tumor cases when accidentally detected are [11,12]. The North American Neuroendocrine Tumor Society guidelines state that observation is appropriate for patients with NF PNENs smaller than 1 cm. Still, the management of 1 to 2 cm lesions should be individualized based on age, comorbidities, growth, grade, the extent of the surgical procedure needed, and patient preference [28]. Massironi et al. followed 51 patients with PNENs, 15 being small NF PNENs, as per ENETS guidelines without surgery [45]. All 15 people were followed up with for four years and survived, with only one developing the disease. The “watch-and-wait” method is stated to be safe in cases of small PNENs with a high solubility and low grade. Sallinen et al. ran a meta-analysis of nine previous papers, and found that when 344 patients with small NF PNENs were followed up with for 32–45 months, the tumors grew in 22%, metastasis was observed in 0%, and surgery was performed in 12% of patients [46]. Sallinen et al. suggested the “watch-and-wait” method rather than surgery based on their analysis considering the low tumor growth, the low need for surgery, and the very low possibility of metastasis. Barenboim et al. observed 44 patients with small asymptomatic, biopsy-proven low-grade PNETs with a Ki-67 proliferative index of <3% for a mean of 52.48 months [47]. Gallium^67^ DOTATOC-PET scans completed in 32 patients demonstrated an uptake in pancreatic tumors in 25 (78%) of those patients. No patient developed systemic metastases. Two patients underwent resection due to tumor growth, and true tumor enlargement was evidenced in the final pathology in one of them. Fifty-five patients underwent immediate resection and were followed up with for 52.8 months. In surgically treated patients, the operative complication rate was significant, and included Clavien–Dindo grade 3–4 complications in 18% of patients and one case of perioperative mortality (1.8%). Therefore, the authors concluded that the expectant management of patients with small, asymptomatic, biopsy-proven low-grade PNETs is safe [47].

### 3.2. Surgery

The surgical management of PNENs is indicated for NF tumors of ≥2 cm, functional tumors, symptomatic, or having evidence of a local invasion or lymphatic metastasis. However, there are reports that surgery should be performed even in patients with small NF, because they can be aggressive in behavior and metastasize. Sharpe et al. analyzed 380 cases of small NF PNENs in patients through the U.S. cancer database [48]. However, 81% of patients underwent surgery, and 19% were followed-up with without surgery. The surgical group’s 5-year survival rate was 82.2%, whereas, in the follow-up group, it was 34.3%. The surgical group showed a higher survival advantage than the follow-up group. Finkelstein et al. performed a meta-analysis of 714 patients with small PNENs from 11 reported papers. However, of the 714,587 patients that underwent surgery, 127 were followed up with [49]. Comparing the survival rates at 1, 3, and 5 years, the surgical group had improved survival rates compared with the follow-up group. With the surgical method, a simple extraction had a shorter surgical time and less bleeding than conventional surgery, but those who underwent pancreatic fistula did well after surgery. Recently, Zhu et al. analyzed patients with NF PNENs between 1 and 2 cm using the surveillance, epidemiology, and end results (SEER) database [50]. After propensity score matching, there were 106 patients in the observation group and 183 patients in the resection group. Patients undergoing resection were associated with a similar 5-year cancer-specific survival, but a longer 5-year overall survival than those under observation [50]. Older patients (≥60 years) can benefit from surgery, but the treatment of younger patients should be individualized. Since pancreatic surgery is still a treatment with a high morbidity and mortality, the location of the tumor, comorbid disease, and surgical risk should also be fully considered in determining the treatment policy of patients with small PNENs. Beane et al. recently suggested the enucleation of small PNENs, which offers several advantages over formal resection, including reductions in operative time, transfusions, the development of postoperative complications, and shortened postoperative length of stay [51].

#### Lymph Node Dissection

In tumors larger than 2 cm, the National Comprehensive Cancer Network (NCCN) guidelines recommend formal resection with lymphadenectomy. However, there is no consensus for smaller tumors [52]. Additionally, regional lymphadenectomy may result in the inclusion of splenectomy, more blood loss, longer operation and recovery time, and a high risk of developing lymphocele [53]. Thus, the benefits and risks of lymphadenectomy should be carefully evaluated. A recent multicenter international study found that in resected sporadic NF PNENs, the risk of lymph node metastases was correlated with tumor size [19]. The decision to undertake surgery in this subgroup of patients should be tailored to each patient’s surgical fitness level, because spontaneous NF PNENs between 1.1 and 2 cm had a higher probability of lymph node metastasis and recurrence than tumors 1 cm or less [19].

### 3.3. EUS-Guided Treatment

Recently, studies on treating small PNENs using ethanol cauterization or radiofrequency ablation (RFA) under EUS have been published, which can be used in high-risk groups [54,55,56]. Ten patients, seven with NF PNENs, one with insulinoma, and two with solid-pseudopapillary neoplasms, were treated with EUS-guided RFA [57]. In seven (70%) of the patients, a complete radiologic response was observed at their 13-month follow-up. Procedure-related adverse events were observed in 12.4% of patients, including one episode of acute pancreatitis. Three patients with symptomatic insulinoma underwent EUS-guided RFA, showed immediate clinical and biochemical improvements within 48 h after the procedure, and maintained a good response at their 12-month follow-up [58]. In a retrospective multicenter study, EUS-guided RFA was evaluated for treating 27 lesions with a mean size of 14.37 ± 7.3 mm in 11 patients with NF PNENs and 7 patients with insulinoma [59]. A complete radiological response was reported in 17 of the 18 patients. No clinically significant recurrences were seen at a mean follow-up period of 8.7 months. Younis et al. also demonstrated that EUS-guided RFA was feasible and well-tolerated in patients with PNENs [60].

Recently, So et al. reported on a propensity score-matching study of EUS-guided ethanol ablation and surgical resection for patients with small NF PNENs [61]. A total of 89 matching patients was included in the EUS-guided ethanol ablation and surgery groups. Early major complications were less frequent in patients who underwent EUS-guided ethanol ablation compared with surgery (0% vs. 11.2%; *p* = 0.003). Late major complications occurred more frequently after surgery, although there was no significant difference between the groups (3.4% vs. 10.1%, *p* = 0.07). The 10-year overall and disease-specific survival rates for both treatments were comparable.

### 3.4. Follow-Up of Patients with PNENs

According to NCCN recommendations, a clinical assessment using biomarkers and CT/magnetic resonance imaging (MRI) should be conducted 3 to 12 months following surgery, with reviews then conducted every 6 to 12 months for a maximum of 10 years [62]. According to ENETS recommendations, patients with G1 or G2 PNETs need to be monitored every 3 to 9 months with the measurement of biochemical markers and standard imaging, such as CT and MRI. Clinical exams and standard imaging should be conducted every 3 months for the first 2 to 3 years following surgical resection, and every 6 to 12 months for the next 5 years in patients with localized R0/R1 resected G3-PNETs. Clinical exams and standard imaging should be performed on patients with the advanced disease every 2 to 3 months while on active therapy [25].

## 4. Conclusions

In managing patients with small PNENs of less than 2 cm, the guidelines need to be revised so that the tumor size can be classified into more subdivisions and the appropriate treatment policy can be determined. Based on recent studies, the “watch-and-wait” method may be strongly recommended for patients with PNENs of less than 1 cm, and the criteria for patients with small PNENs may be lowered from 2 cm to 1.5 cm or less. The location of the tumor, concomitant conditions, and surgical risk should all be taken into account when deciding the treatment for patients with small PNENs, because pancreatic surgery is still a treatment with a high morbidity and mortality. In addition, it is recommended to actively conduct an EUS-guided biopsy on patients with small PNENs to evaluate the potential risk of malignancy. In particular, when atypical PNENs are observed in imaging findings, it would be helpful to perform an EUS-guided biopsy to confirm the PNEN and evaluate the risk of malignancy. In patients who need surgical resection but are not suitable for surgery, new treatments, such as ethanol cauterization or RFA, can be attempted under EUS. A large, randomized prospective study is needed to determine whether a follow-up is better or surgery is better in patients with small PNENs.

## Figures and Tables

**Table 1:** The World Health Organization 2017 classification for neuroendocrine neoplasms.

NET-G1	NET-G2	NET-G3	NEC-G3
Well-differentiated Mitotic count < 2/2 mm^2^ Ki-67 ≤ 2%	Well-differentiated Mitotic count < 2–20/2 mm^2^ Ki-67 3–20%	Well-differentiated Mitotic count > 20/2 mm^2^ Ki-67 > 20%	Poorly differentiated Mitotic count > 20/2 mm^2^ Ki-67 > 20%

NET: neuroendocrine tumor; NEC: neuroendocrine carcinoma.

## Data Availability

Not applicable.
